# Supplementary figures and images for: Rethinking the use of finite element simulations in comparative biomechanics research
Source: PeerJ. 2021 Apr 7;9:e11178. doi: 10.7717/peerj.11178 (PMC8035905; doi:10.7717/peerj.11178)

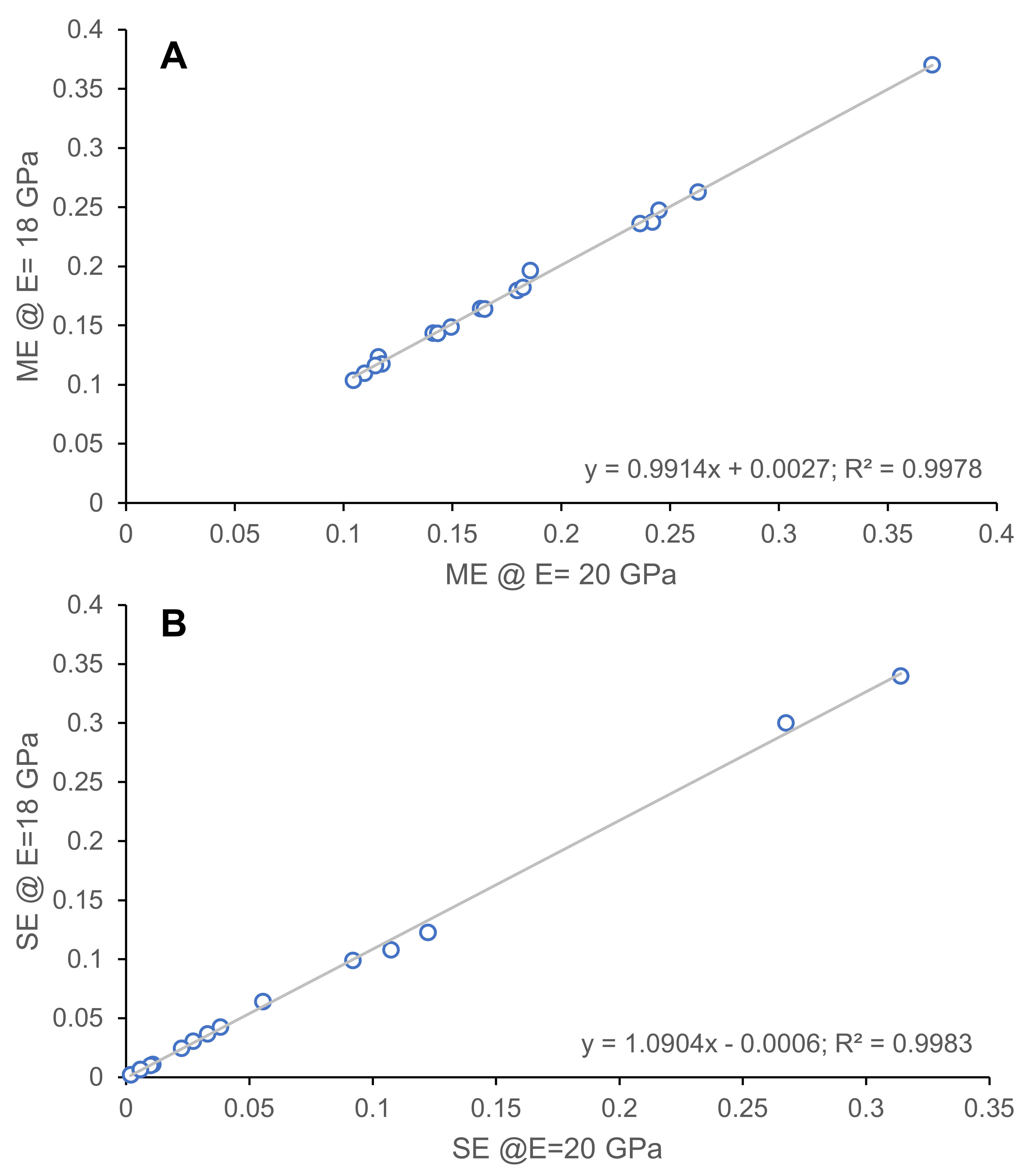

Supplement: Supplemental Information 2 — A. Mechanical efficiency values, B. Strain energy values. [file peerj-09-11178-s002.png]
